# Enhancing nursing documentation in Kazakhstan: assessing utilization and standardization for improving patient care

**DOI:** 10.3389/fpubh.2023.1267809

**Published:** 2023-11-23

**Authors:** Bibinur Sydykova, Dariga Smailova, Zaituna Khismetova, Marzhan Brimzhanova, Zaure Baigozhina, Hengameh Hosseini, Natalya Latypova, Marina Izmailovich

**Affiliations:** ^1^Semey State Medical University, Semey, Kazakhstan; ^2^Kazakh National Medical University, Almaty, Kazakhstan; ^3^Department of Health Economics and Insurance Medicine, Kazakhstan Medical University “KSPH”, Almaty, Kazakhstan; ^4^Astana Medical University, Nur-Sultan, Kazakhstan; ^5^University of Scranton, Scranton, PA, United States; ^6^Karaganda State Medical University, Karaganda, Kazakhstan

**Keywords:** nursing documentation, adherence, attitude towards documentation, nurses knowledge, standard operating procedure

## Abstract

**Background and aim:**

This article stresses the importance of comprehensive nursing documentation in scientific medicine and discusses the adoption of standardized terminologies in Europe. The study also presents findings from a cross-sectional study conducted in Kazakhstan, assessing the utilization of standard operating procedures and nursing documentation in various clinical scenarios. The aim was evaluate the level of use of the form of nursing documentation and Standard Operating Procedure within the framework of reforming the Republic of Kazakhstan.

**Materials and methods:**

During the period from December 2021 to February 2022, a cross-sectional study was conducted in Kazakhstan, involving a randomly selected sample of nurses with technical and vocational education as well as those with applied/academic baccalaureate degrees in nursing.

**Results:**

In this cross-sectional study of 2,263 female nurses, 75.3% were nurse practitioners, and 44% held the highest qualification category. Awareness levels varied, with around 64.7% aware of the pilot program for care services, 65.8% aware of the deputy head position, and 73.8% familiar with the “extended practice nurse” role. Only 55.2% knew about the International Clinical Nursing Classification, and 54.5% observed changes in their nursing approach due to education. The limb edema measurement checklist was not used by the majority (88.4%) of respondents, and 68% did not utilize the antibiotic susceptibility testing checklist. Various other checklists and algorithms had limited utilization, with percentages ranging from 9.1 to 69.3%, indicating varying levels of adoption among participants. For assisting children with cerebral palsy, the “Assessment of hand use capacity according to the MACS classification system” was utilized by 9.1%, while 90.9% did not employ it. In the context of communication, 30.7% of the respondents utilized the “Algorithm of actions of a medical registrar when communicating with a patient,” while 69.3% did not use it. These findings highlight variable adoption rates among participants for these medical procedures and protocols.

**Conclusion:**

In Kazakhstan, nursing documentation forms and Standard Operating Procedures face challenges and limited utilization, but their implementation has shown positive impacts on patient care and healthcare outcomes. Overcoming resistance to change, increasing awareness, and addressing resource constraints are essential for further improvement.

## Introduction

Comprehensive medical documentation, particularly nursing documentation, is crucial for patient care continuity and legal purposes (1). The nursing process involves systematic steps, including assessment, problem identification, goal setting, intervention formulation, and execution (2–4). Nursing documentation has been essential since Florence Nightingale and serves various roles in contemporary healthcare, including continuity of care and legal evidence (5). In Europe, the adoption of standardized nursing terminologies varies, with NNN terminologies widely used but posing challenges for cross-country comparisons. Efforts to standardize nursing language have been ongoing since 1995 through the Association for Common European Nursing Diagnoses, Interventions, and disease outcomes (6). Emphasizing evidence-based practice is crucial for bridging the gap between research and practice, improving patient outcomes, and enhancing healthcare quality while managing costs (7).

The Perioperative Nursing Data Set (PNDS) offers a functional tool for documenting patient information in perioperative care, differing from other classification systems like NANDA International Inc., ICNP, or locally developed electronic health records (8). As healthcare evolves into a digital age, personalized and precise healthcare is expected to improve outcomes and cost-effectiveness, reducing unnecessary mass screenings and treatments (9). Increasing medical complexity and patient-centered care challenge clinical nurses, emphasizing the importance of critical thinking for patient safety and high-quality care (10). The American Nursing Association (ANA) encompasses all aspects of nursing care, and deficiencies in nursing documentation quality are attributed to negative attitudes and knowledge gaps (11). Notably, the quality of electronic-based nursing care is not significantly impacted by education level, but lower-educated nurses may seek further education to enhance hospital services. Higher nurse education levels are associated with improved electronic nursing documentation performance (12).

In acute care settings, inadequate nursing documentation can harm patient outcomes and even lead to legal action, underscoring the need for strategies to enhance its quality (13, 14). Nurse Practitioners have the authority to diagnose, prescribe medication, and order treatments (15), with their role originating in the late 1960s in the United States (16) and late 1970s in Canada (17, 18). Moving beyond nursing documentation requires a shift in mindset, recognizing Electronic Health Records (EHRs) as databases, necessitating accurate, relevant, and timely data in the digital age (9). Scientific literature suggests that optimal hospital nurse staffing and higher nursing education levels, such as bachelor’s degrees, are associated with lower mortality rates (19). However, understanding the causal mechanisms behind these associations remains a challenge, as nursing is often targeted for cost savings, making efficiency improvements more difficult to achieve (20).

Kazakhstan is currently in the early stages of implementing global nursing standards, and there have been no studies conducted to assess the current status of this implementation. As the country progresses in adopting these nursing standards, further research will be essential to evaluate their effectiveness and impact on healthcare practices. The aim of this study was to evaluate the level of utilization of nursing documentation forms and Standard Operating Procedures (SOPs) within the framework of healthcare reform in the Republic of Kazakhstan.

## Materials and methods

### Study design and procedures

Overall, there were 2,263 respondents in this cross-sectional study. During the period between December 2021 and February 2022, a cross-sectional study was conducted in Kazakhstan, involving nurses who (1) graduated from medical college (have a technical or vocational education), (2) graduated from a medical university (have a higher education), and (3) completed an applied baccalaureate (equivalent to higher education). The applied baccalaureate is a special 2-year program at a university for nurses who have graduated from college. Upon completion of this program, nurses receive a baccalaureate degree and are equivalent to academic baccalaureates. We used convenience sampling for selecting respondents for the study. Inclusion criteria were voluntary participation in the study, being practical health care nurses and graduates medical college or university. Exclusion criteria were refusal to participate in the study and specialists who did not graduate from higher education institutions. As per the Center for Development of Education and Science, the estimated number of working nurses in 2021 exceeded 175,000. The sample size for the study was determined using the StatCalc-Sample Size and Power calculator [EPI Info], taking into account the population size of 175,000 nurses, an anticipated frequency of 99.99%, a margin of error of 5%, and an estimated effect size of 1.0. Based on these parameters, the calculated sample size was 1,501. However, to account for potential dropouts, a total of 2,277 participants were enrolled. Ultimately, 2,263 nurses agreed to participate in the final online survey, resulting in a response rate of 96.9%.

### The tool and data collection techniques

The self-developed questionnaire consisted of 27 questions. The first section included questions related to respondents’ social and demographic characteristics, qualifications, knowledge of nursing programs, and nursing practice. Data were analyzed using descriptive statistics such as averages and percentages. Informed consent was sent to responders with survey link, and respondents’ completed questionnaire form was equated to consent for the study. The questionnaire was completed in approximately 20–25 min. No personal information was collected in order to maintain confidentiality. All data were encrypted and stored electronically in a secure location, and the password was only available to the principal investigator (PI) to ensure confidentiality of study participants.

### Statistical analysis

The study data were analyzed using the Statistical Package for the Social Sciences (SPSS) version 20. Categorical variables were presented as frequencies in percentages. To assess the association between categorical variables, Pearson’s chi-square test was used. A *p*-value of 0.05 or lower was considered statistically significant.

### Ethical considerations

The Ethics Committee of Semey Medical University with registration No 2, dated 28.10.2020, has approved this study.

## Results

Out of 2,263 respondents 3.6% were male and 96.4% were female. 75.3% of respondents are nurse practitioners, 2.8% are specialized nurses. The highest qualification category has 44% of respondents (Table 1).

**Table 1:** General characteristics of the study participants (*n* = 2,263).

Variables		*n*	%	*p*-value
Gender:	Males	82	3.6	≤0.001
	Female	2,181	96.4	
Age (years)	*M* (*SD*)	40.16	12.37	
	Min	18		
	Max	66		
	CI 95%	39.65–40.67		
Position:	General practice nurse	1703	75.3	≤0.001
	Advanced practice nurse	63	2.8	
	Consultant Nurse Specialist Nurse	328	14.5	
	Head Nurse	142	6.3	
	Head Nursing Sister	22	1.0	
	Deputy Director of Nursing	4	0.2	
	Centre Director of nursing	1	0.0	
Qualification:	Higher	996	44.0	≤0.001
	First	314	13.9	
	Second	188	8.3	
	No category	765	33.8	

Data provides insights into participant awareness across several areas: 64.7% were aware of the pilot program for care services development, 65.8% knew about the introduction of the deputy head position, and 73.8% were familiar with the “extended practice nurse” role in Kazakhstan. Additionally, 81.9% opposed the introduction of a coordinator of nursing services at the regional health department, and 68% were aware of nursing tariffs. Most notably, 62.3% of participants did not receive extra payment for extended services, and 72.9% considered nursing documentation necessary (Table 2).

**Table 2:** Awareness and knowledge of nursing program developments and guidelines *(N* = 2,263).

Component	Yes	No
Is your organization a pilot organization for the development of nursing services?	1,465 (64.7%)	798 (35.3%)
Did you know that Kazakhstan has introduced the position of “deputy head of nursing”?	1,488 (65.8%)	775 (34.2%)
Are you aware that the position of “extended practice nurse” has been introduced in Kazakhstan?	1,670(73.8%)	593(26.2%)
Do you think it is right to introduce the position of a coordinator of nursing services in the regional health department?	1854(81.9%)	409 (18.1%)
Ministry of Health of the Republic of Kazakhstan Order M-199/2020 of 23 November 2020 No. KP DSM On approval of the rules of nursing care (Article 127(6) of the RK Code of 7 July 2020 on Public Health and the Health Care System).		
Did you know that nursing fees have been introduced in Kazakhstan?	1,538 (68.0%)	725 (32.0%)
Do you receive extra pay for extended services?	853 (37.7%)	1,410 (62.3%)
Is there a need for nursing documentation?	1,650 (72.9%)	613 (18.1%)
Clinical Care Classification		
Did you know that Kazakhstan has adapted the international Clinical Care Classification (CCC) for nursing practice?	1,249 (55.2%)	1,014 (44.8%)
Do you read international scientific publications on nursing care and organization of nursing care?	1,170 (51.7%)	1,093 (48.3%)
Do you do nursing appointments for patients?	1715 (75.8%)	548 (24.2%)
Have you changed your approach to nursing since completing your applied/academic bachelor’s degree?	1,233 (54.5%)	1,030 (45.5%)
Are you aware that clinical nursing guidelines have been developed and approved in Kazakhstan?	1,553(68.6%)	710 (31.4%)
Do you keep nursing records?	2097 (92.7%)	166 (7.3%)

Among those who did not utilize the limb edema measurement checklist, the majority (88.4%) reported not using it. For the urinary catheter placement checklist, 82% of the respondents did not employ it, whereas 18% reported its usage. The majority (64.5%) of participants did not use the checklist for “Nurse’s tactics on admission of pregnant/maternity/hemorrhage patient,” while 35.5% reported its usage. Similarly, 64.5% of the respondents did not utilize the checklist for “Performing pulse oximetry,” and 35.5% reported its usage. Regarding the “Care of patients with bronchial asthma” checklist, 81% of the participants did not use it, while 19% reported its usage. Lastly, the majority (90%) did not use the checklist for “Care of a patient with non-infectious gastroenteritis and colitis (age: 0–18 years),” while 10% reported its usage (Table 3).

**Table 3:** Which nursing documentation (checklists) do you use during the initial nursing visit? (*N* = 2,263).

IBC Name	Check list	Yes (%)	No (%)	Chi-square	*p*-value
R60.1 (Unspecified oedema)	Measurement of limb swelling	263 (11.6%)	2000 (88.4%)	1,333,261	<0.001
Z96.0 (Urinary catheter placement)	Placement of urinary catheter	407 (18%)	1856 (82%)	927,795	<0.001
O46 (Bleeding from uterus in early pregnancy) O67.9 (Unspecified hemorrhage during and after labor)	Nurse tactics on admission of pregnant/maternity/maternity patient with hemorrhage	804 (35.5%)	1,456 (64.5%)	189,582	<0.001
R03.0 (Painless breathing)	Taking a pulse oximeter	804 (35.5%)	1,459 (64.5%)	189,582	<0.001
J45 (Bronchial asthma)	Care of the patient with bronchial asthma	430 (19%)	1833 (81%)	1,446,081	<0.001
K52.9 (Other specific non-infectious gastroenteritis and colitis)	Care of a patient with non-infectious gastroenteritis and colitis (age: 0–18 years)	227 (10%)	2036 (90%)	869,823	<0.001

The utilization of specific documentation (checklists) during the first patient visit as reported by the respondents. The majority (68%) did not utilize the antibiotic susceptibility testing checklist, while 32% reported its usage. Regarding the checklist for “Reception and care of COVID-19 in children and adolescents,” 75.7% of the participants did not use it, while 24.3% reported its usage. For the checklist on “Nursing reception and nursing care at the first visit of a patient with signs of ARVI, including COVID-19,” 67.8% of the respondents did not utilize it, whereas 32.2% reported its usage. The majority (85.6%) did not use the checklist for “Feeding a critically ill patient through a nasogastric tube,” while 14.4% indicated its usage. Regarding the pressure ulcer management checklist, 77.6% of the participants did not utilize it, whereas 22.4% reported its usage. Similarly, the majority (77.1%) did not use the checklist for “Oxygenation with nasal cannula or oxygen mask (oxygen therapy),” while 22.9% reported its usage (Table 4).

**Table 4:** Which nursing documentation (checklists) do you use during the initial nursing visit? (*N* = 2,263).

IBC Name	Check list	Yes (%)	No (%)	Chi-square	*p*-value
Z16.2 (Examination for infection and infectious disease)	Antibiotic sensitivity testing	724 (32%)	1,539 (68%)	293,515	<0.001
U07.1 (COVID-19, confirmed by laboratory)	COVID-19 nursing and nursing care for children and adolescents	549 (24.3%)	1714 (75.7%)	599,746	<0.001
Z20.8 (Contact with sources of infectious and parasitic diseases, other specified)	Nursing and nursing care for initial clinic/filter visit with signs of acute respiratory infection, including COVID19	728 (32.2%)	1,535 (67.8%)	287,781	<0.001
Z93.1 [Artificial respiration (permanent tracheostomy)]	Feeding the critically ill patient via nasogastric tube	325 (14.4%)	1938 (85.6%)	1,149,699	<0.001
L89.9 (Bedsores, unspecified)	Treating bedsores	506 (22.4%)	1757 (77.6%)	691,560	<0.001
Z93.6 (Artificial feeding)	Administration of oxygen through a nasal cannula or an oxygen mask (oxygen therapy)	519 (22.9%)	1744 (77.1%)	663,113	<0.001
	MACS (Manual Ability Classification System) assessment of hand use in children with cerebral palsy		2,263 (100%)		

The frequency of utilization of standard operating procedures among the respondents. The diabetic foot care algorithm for “Hygiene treatment of feet in diabetic patients” was utilized by 26.5% of the participants, while 73.5% did not use it. The examination for “Foot vibration sensitivity in diabetic patients” was used by 13.6%, with 86.4% not using it. The “Foot vascularity study in diabetic patients” was utilized by 11.1% of the respondents, while 88.9% did not utilize it. Similarly, the examination for “Foot tactile sensitivity in diabetic patients” was used by 11% of the participants, with 89% not using it. For “Foot temperature sensitivity examination in diabetic patients,” 12.4% reported its usage, while 87.6% did not utilize it. The “Foot examination in diabetic patients” was utilized by 12.3% of the respondents, while 87.7% did not employ it (Table 5).

**Table 5:** Utilization of standard operating procedures in various clinical scenarios.

Standard operating procedures (SOP)	Yes	No	Chi-square	*p*-value
Diabetic foot care algorithm				
SOP “Hygiene treatment of feet in diabetic patients”	600(26.5%)	1,663(73.5%)	1,382,837	<0.001
Study of foot vibration sensitivity in diabetic patients	308(13.6%)	1955(86.4%)	1,507,472	<0.001
Study of foot vascularity in diabetic patients	252 (11.1%)	2011 (88.9%)	1,367,247	<0.001
Study of foot tactile sensitivity in diabetic patients	250 (11.0%)	2013(89.0%)	1,373,473	<0.001
Feet sensitivity study in diabetic patients	281 (12.4%)	1982 (87.6%)	1,278,569	<0.001
Foot examinations in diabetic patients	279 (12.3%)	1984 (87.7%)	1,389,098	<0.001
Emergency obstetric and gynaecological care				
OARIT Nurse management of severe pre-eclampsia and eclampsia	411 (18.2%)	1852 (81.8%)	1,411,122	<0.001
Nurse management of a pregnant and postpartum woman on admission with hemorrhage	434 (19.2%)	1829 (80.8%)	859,932	<0.001
Blood pressure investigations				
Measure patient’s blood pressure	1,409 (62.3%)	854 (37.7%)	136,114	<0.001
Measure patient’s pulse on the radial artery	565 (25.0%)	1,698 (75%)	567,251	<0.001
Recognition of White Coat Hypertension by Advanced Practice Nurse	356 (15.7%)	1907 (84.3%)	1,063,014	<0.001
Interventions for patients with respiratory conditions				
Conducting a computerized spirography	203 (9.0%)	2060 (91.0%)	1,523,840	<0.001
Restorative breathing exercise	443 (19.6%)	1820 (80.4%)	837,883	<0.001

Regarding obstetric and gynecological emergencies, the “Emergency OARIT Nurse Tactics for Severe Preeclampsia and Eclampsia” was used by 18.2% of the participants, whereas 81.8% did not utilize it. The “Nurse Tactics for Obstetric Bleeding in Pregnant Women” was used by 19.2, and 80.8% did not employ it. In the screening for arterial hypertension, “Taking patient’s blood pressure” was used by 62.3% of the respondents, while 37.7% did not utilize it. The examination for “Checking the patient’s pulse on the radial artery” was used by 25% of those who utilized it, and 75% did not use it. The “Recognition of White Coat Hypertension by Extended Practice Nurse” was utilized by 15.7% of the respondents, while 84.3% did not employ it. Computer spirometry was used by 9%, compared to 91% of non-users, for activities performed on patients with respiratory diseases. The utilization rate of “Restorative breathing exercises” was 19.6%, while 80.4% did not utilize it (Table 5).

The frequency of utilization of standard operating procedures among the respondents for various clinical scenarios. In the management of patients with chronic heart failure, “Measuring the body weight of a patient with CHF” was used by 36.1% of the participants, while 63.9% did not utilize it. Self-care training for patients with CHF was utilized by 20.9% of the respondents, and 79.1% did not employ it. The procedure of “Daily diuresis and assessment of water balance in CHF” was used by 18.6% of the participants, with 81.4% not using it. For “Counselling a CCF patient in need of palliative care,” 12.8% of the respondents utilized it, while 87.2% did not employ it. Similarly, the procedure of “Monitoring the condition of a patient with CHF at the end of life” was used by 10.5% of the participants, and 89.5% did not utilize it. Providing individualized care for a patient with end-stage CHF was utilized by 10.8, and 89.2% did not utilize it (Table 6).

**Table 6:** Utilization of standard operating procedures in the management of patients with chronic heart failure, cerebral palsy and stroke.

Standard operating procedures (SОP)	Yes	No	Chi-square	*p*-value
Management of a patient with chronic heart failure				
Measuring the patient’s body weight in CHF	817 (36.1%)	1,446 (63.9%)	174,830	<0.001
Self-care education for the cardiac patient	472 (20.9%)	1791 (79.1%)	768,785	<0.001
Determining daily diuresis and water balance in CHF	422 (18.6%)	1841 (81.4%)	889,775	<0.001
Counselling the CCN patient who requires palliative care	289 (12.8%)	1974 (87.2%)	1,254,629	<0.001
Monitoring the ICF patient at the terminal stage	238 (10.5%)	2025 (89.5%)	1,411,122	<0.001
Providing individual care for a terminally-ill cardiac patient	245 (10.8%)	2018 (89.2%)	1,389,098	<0.001
Algorithm of actions by medical registrar when communicating with a patient	695 (30.7%)	1,568 (69.3%)	336,778	<0.001
How to manage an aggressive or distressed patient	444 (19.6%)	1819 (80.4%)	835,451	<0.001
Support for children with cerebral palsy				
Assessment of hand use according to the MACS classification system in children with cerebral palsy	206 (9.1%)	2057 (90.9%)	1,514,008	<0.001
CFCS Communication Function Classification System (CFCS) assessment in children with cerebral palsy	290 (12.8%)	1973 (87.2%)	1,251,652	<0.001
Eating and Drinking Ability Classification System (EDACS) assessment in children with cerebral palsy	121 (5.3%)	2,142 (94.7%)	1804,879	<0.001
Rehabilitation after a stroke				
Recovery of motor skills in patients with cerebral stroke	247 (10.9%)	2016 (89.1%)	1,382,837	<0.001
Preventing injuries and falls	740 (32.7%)	1,523 (67.3%)	270,919	<0.001
Cognitive rehabilitation of post-stroke patients	224 (9.9%)	2039(90.1%)	1,382,837	<0.001
Physical therapy for stroke patients	279 (12.3%)	1984 (87.7%)	1,284,589	<0.001
Mindfulness therapy for stroke patients	208(9.2%)	2055 (90.8%)	1,507,472	<0.001
Physical activity evaluation of the patient	433 (19.1%)	1830 (80.9%)	862,399	<0.001

In the context of communication, 30.7% of the respondents utilized the “Algorithm of actions of a medical registrar when communicating with a patient,” while 69.3% did not use it. The “Rules of interaction with an aggressive or stressed patient” were utilized by 19.6% of the participants, with 80.4% not utilizing it.

For assisting children with cerebral palsy, the “Assessment of hand use capacity according to the MACS classification system” was utilized by 9.1%, while 90.9% did not employ it. The utilization of the Communication Function Classification System (CFCS) assessment in children with cerebral palsy was reported by 12.8% of the respondents, while 87.2% did not utilize it. Similarly, the utilization of the Eating and Drinking Capacity Classification System (EDACS) for children with cerebral palsy was reported by 5.3, and 94.7% did not utilize it.

In the context of post-stroke rehabilitation, the Motor Activities Recovery for Cerebral Stroke Patients method was utilized by 10.9% of the participants, and 89.1% did not utilize it. The “Injury and fall prevention” method was utilized by 32.7% of the respondents, while 67.3% did not utilize it. The utilization of “Cognitive rehabilitation for post-stroke patients” was reported by 9.9%, whereas 90.1% did not utilize it. The utilization of physiotherapy for stroke patients was reported by 12.3% of the participants, while 87.7% did not utilize it. Additionally, the utilization of “Mirror therapy for stroke patients” was reported by 9.2%, with 90.8% not utilizing it. Lastly, the assessment of a patient’s physical activity level was utilized by 19.1% of the respondents, while 80.9% did not utilize it.

## Discussion

Thus, our study was the first in Kazakhstan to examine the reform of nursing documentation. In connection with the step-by-step reform of nursing services in Kazakhstan, the experience of Finnish experts in nursing management is being adopted. The positions of “extended practice nurse,” “deputy director of nursing” were introduced, as well as the concepts of “nursing diagnosis,” “nursing intervention,” “nursing outcome,” and “nursing documentation” at the legislative level of the country. The organizational structure was changed and the staffing table was amended to introduce new positions in nursing. Clinical nursing guidelines, standards of operating procedures, and nursing documentation forms were developed.

Based on the analysis of international experience, different countries have adopted various approaches to nursing documentation. European countries learn from each other’s experiences by implementing minimum sets of nursing data based on the International Classification of Nursing Practice. Therefore, during the reform of the nursing service in the Republic of Kazakhstan, it is important to introduce an adapted version of the Clinical Care Classification that is suitable for the country’s healthcare system. In Kazakhstan, the National Project for the implementation of a new nursing service model has been underway since 2018. In 2020, the International Classification of Nursing Practice has been adopted but not yet officially approved at the national level. The benefits of the new nursing service model in the Republic of Kazakhstan are the development of nurses’ skills according to the level of qualification (8 levels). Each degree in nursing or certification in a specialized nursing program offers new opportunities for career advancement. Also, the implementation of evidence-based medicine has stimulated nursing: nurses have to be more efficient in patient care and make decisions based on proven facts.

As reported in a literature review from 2007, the electronic nursing records in Finland lacked standardization and were not interconnected with the patient’s medical history. However, the introduction of a unified and standardized nursing record at a national level has addressed this issue. This standardized nursing record incorporates standardized nursing data, enabling the effective management and evaluation of the nursing process. Furthermore, it facilitates the integration of the nursing record with the comprehensive patient history maintained by the multidisciplinary healthcare team (13).

The findings of our survey indicate that a significant proportion of nurses had knowledge regarding several key developments in the healthcare system. A majority of the respondents were aware of the introduction of the deputy manager position (65.8%), the extended practice nurse position (73.8%), and the nursing guide (68%). However, it is worth noting that only 55.2% of the participants were aware that Kazakhstan has implemented the International Classification of Nursing Practice, specifically the “Classification of Clinical Nursing.”

On the topic of the introduction of a nursing service coordinator in the regional health department, the majority of respondents (81.9%) expressed support for this initiative, considering it to be appropriate and beneficial. It is consistent with the study of Saranto and Kinnunen, who performed the evaluation of nursing documentation, revealing predominantly positive outcomes in terms of quality, adherence to the nursing process, utilization of standardized terminology, level of knowledge, and acceptance of computerized documentation (21). Similar results were obtained in Brazil, where the authors investigated the utilization of Standard Operating Procedures (SOPs) among nurses. The study population predominantly consisted of nurses (83.91%), specifically women aged 20 to 40 years (79.7%). Among the surveyed healthcare institutions, SOPs were implemented in three of them. All participants acknowledged the presence of SOPs in their respective units and considered compliance with these protocols important. Remarkably, a high percentage (95.4%) expressed no difficulties in comprehending the SOPs. However, some challenges were reported, including difficulties in understanding outdated techniques and a lack of necessary materials. Furthermore, among the nurses, 62.86% believed that not all team members adhered to the SOPs (22).

Regarding nursing practice, our results indicated that a majority of nurses (75.8%) adhered to nursing appointments and 92.7% maintained nursing records. Moreover, 59.3% of the respondents associated the nursing diagnosis with the “diagnosis according to the Clinical Care Classification,” while 40.7% associated it with “ICD-10 diagnosis,” which is more applicable to medical diagnosis. However, it is noteworthy that only 37.7% of the respondents received additional compensation for providing extended services, suggesting that most nurses do not receive additional payment for the additional care they provide. As noted by Chapman, Wides and Spetz, nurses of advanced practice are able to provide as qualified patient care and obtain the same treatment results as physicians, but paid at considerably lower rates (23). We believe that implementation of the adapted international Clinical Care Classification nursing practice classifier at the national level will allow nurses to record nursing interventions/services with subsequent payment for nursing services rendered at the expense of state funding system.

Regarding the impact of education level on nursing practice, more than half of the respondents (54.5%) indicated a change in their approach to nursing after completing an applied/academic bachelor’s degree. Sibandze and Scafide in their systematic review highlighted that nurses holding bachelor or higher degree showed a deeper awareness and application of professional values compared to nurses with non-academic education (24). This demonstrates the importance of academic learning in development of professional skills and therefore in improving quality of nursing care.

However, Mensah et al. observed that certain healthcare institutions faced challenges in implementing specific clinical guidelines, such as universal or daily self-monitoring. Notably, many of these guidelines did not address the potential barriers resulting from resource constraints. Several factors contributing to these challenges were identified, including insufficient training opportunities for nurses, high staff turnover rates, absence of standardized protocols and diagnostic tools, limited availability of consumables and equipment, inadequate funding for healthcare services and treatment (25). In our study, the clinical nursing guidelines most commonly utilized by the respondents were ‘Preventing and treating pressure sores’ (39%) and ‘Preventing falls and reducing injuries caused by falls’ (34.3%). However, there is a notable lack of utilization of checklists during initial nursing appointments, particularly for patients with glaucoma (85.8%) and acute stroke (72.5%). Similar study an overall analysis encompassing knowledge and attitude tests, as well as the evaluation of nursing records was conducted by Leoni-Scheiber, Mayer and Müller-Staub. Their findings revealed a relatively low level of nurses’ knowledge, a positive attitude, and an average quality of advanced nursing processes. Furthermore, 79% nurses responded that they utilize clinical manuals. This suggests that some nurses do not consistently refer to clinical guidelines (26).

Overall, our research underscores the importance of ongoing education and training for nurses in Kazakhstan, particularly in relation to the implementation of international nursing practices and guidelines. Additionally, it emphasizes the necessity for policies that support and encourage the expansion of nursing services. The study’s findings offer valuable insights into the current state of nursing practice in Kazakhstan and can serve as a foundation for future policy decisions and interventions aimed at enhancing nursing practice. Furthermore, it highlights the significance of policies that promote and facilitate the growth of nursing services.

## Limitations

The study utilized a cross-sectional design, which may not provide a representative sample of the entire nursing population in Kazakhstan. The participants were selected from nurses with technical and vocational education as well as those with applied/academic baccalaureate degrees, potentially excluding other categories of nurses and limiting the generalizability of the findings.

The data collected through the questionnaire relied on self-reported responses from the participants. This introduces the possibility of response bias, where participants may provide socially desirable answers or misreport their practices and knowledge regarding nursing documentation and SOPs.

The study focused solely on nursing documentation forms and SOPs within the context of healthcare reform in Kazakhstan. Other factors influencing the utilization of these forms, such as organizational policies, management support, and availability of technological resources, were not thoroughly explored, potentially overlooking additional influential variables.

## Conclusion

The utilization of nursing documentation forms and Standard Operating Procedures (SOPs) in healthcare organizations during the healthcare reform in Kazakhstan is below optimal levels due to various challenges, including resistance to change, limited awareness and training, resource constraints, and organizational culture. However, the implementation of standardized nursing documentation forms and SOPs has shown promising improvements in patient care quality and healthcare outcomes. To improve the situation, recommendations include developing clear guidelines and policies, providing comprehensive training programs for healthcare professionals, allocating adequate resources for integration, fostering a culture of collaboration and continuous improvement, and regularly monitoring and evaluating the effectiveness of these practices. By addressing these tasks, healthcare organizations can overcome barriers and enhance patient care and outcomes through improved utilization of nursing documentation forms and SOPs.

## Data availability statement

The original contributions presented in the study are included in the article/supplementary material, further inquiries can be directed to the corresponding author.

## Author contributions

BS: Conceptualization, Writing – original draft, Data curation, Methodology. DS: Project administration, Writing – original draft, Formal Analysis, Methodology, Data curation. ZK: Methodology, Writing – review & editing. MB: Data curation, Writing – review & editing. ZB: Data curation, Writing – review & editing. HH: Methodology, – review & editing. NL: Writing – review & editing, Conceptualization, Validation. MI: Writing – review & editing, Visualization.
