# Correction: Enhancing nursing documentation in Kazakhstan: assessing utilization and standardization for improving patient care

**DOI:** 10.3389/fpubh.2025.1722480

**Published:** 2025-11-28

**Authors:** Bibinur Sydykova, Dariga Smailova, Zaituna Khismetova, Marzhan Brimzhanova, Zaure Baigozhina, Hengameh Hosseini, Natalya Latypova, Marina Izmailovich

**Affiliations:** 1 Semey State Medical University, Semey, Kazakhstan; 2 Kazakh National Medical University, Almaty, Kazakhstan; 3 Department of Health Economics and Insurance Medicine, Kazakhstan Medical University “KSPH”, Almaty, Kazakhstan; 4 Astana Medical University, Nur-Sultan, Kazakhstan; 5 University of Scranton, Scranton, PA, United States; 6 Karaganda State Medical University, Karaganda, Kazakhstan

**Keywords:** nursing documentation, adherence, attitude towards documentation, nurses knowledge, standard operating procedure

There was a mistake in Tables 3–6 as published. The χ^2^ (chi-square) values appeared to be incorrect by several orders of magnitude across multiple tables (for example, values were reported in the millions instead of in the expected thousands). The corrected Tables 3–6 appear below. The data have been recalculated and verified. No other parts of the article are affected.

**Table 3:** Which nursing documentation (checklists) do you use during the initial nursing visit? (*N* = 2,263).

**IBC Name**	**Check list**	**Yes (%)**	**No (%)**	**Chi-square**	***P*-value**
R60.1 (Unspecified oedema)	Measurement of limb swelling	263 (11,6%)	2000 (88.4%)	1333,261^a^	< 0.001
Z96.0 (Urinary catheter placement)	Placement of urinary catheter	407 (18%)	1856 (82%)	927,795^a^	< 0.001
O46 (Bleeding from uterus in early pregnancy)	Nurse tactics on admission of pregnant/maternity/maternity patient with hemorrhage	804 (35.5%)	1456 (64.5%)	189,582^a^	< 0.001
O67.9 (Unspecified hemorrhage during and after labour)					
R03.0 (Painless breathing)	Taking a pulse oximeter	804 (35.5 %)	1459 (64.5%)	189,582^a^	< 0.001
J45 (Bronchial asthma)	Care of the patient with bronchial asthma	430 (19%)	1833 (81%)	1446,081^a^	< 0.001
K52.9 (Other specific non-infectious gastroenteritis and colitis)	Care of a patient with non-infectious gastroenteritis and colitis (age: 0–18 years)	227 (10%)	2036 (90%)	869,823^a^	< 0.001

The letter “a” indicates statistically significant differences between the compared groups (*p* < 0.05).

**Table 4:** Which nursing documentation (checklists) do you use during the initial nursing visit? (*N* = 2,263).

**IBC name**	**Check list**	**Yes (%)**	**No (%)**	**Chi-square**	***P*-value**
Z16.2 (Examination for infection and infectious disease)	Antibiotic sensitivity testing	724 (32%)	1539 (68%)	293,515^a^	< 0.001
U07.1 (COVID-19, confirmed by laboratory)	COVID-19 nursing and nursing care for children and adolescents	549 (24.3%)	1714 (75.7%)	599,746^a^	< 0.001
Z20.8 (Contact with sources of infectious and parasitic diseases, other specified)	Nursing and nursing care for initial clinic/filter visit with signs of acute respiratory infection, including COVID19	728 (32.2%)	1535 (67.8%)	287,781^a^	< 0.001
Z93.1 (Artificial respiration [permanent tracheostomy]	Feeding the critically ill patient via nasogastric tube	325 (14.4%)	1938 (85.6%)	1149,699^a^	< 0.001
L89.9 (Bedsores, unspecified)	Treating bedsores	506 (22.4%)	1757 (77.6%)	691,560^a^	< 0.001
Z93.6 (Artificial feeding)	Administration of oxygen through a nasal cannula or an oxygen mask (oxygen therapy)	519 (22.9%)	1744 (77.1%)	663,113^a^	< 0.001
	MACS (Manual Ability Classification System) assessment of hand use in children with cerebral palsy		2263 (100%)		

The letter “a” indicates statistically significant differences between the compared groups (*p* < 0.05).

**Table 5:** Utilization of standard operating procedures in various clinical scenarios.

**Standard operating procedures (SOP)**	**Yes**	**No**	**Chi-square**	***P*-value**
**Diabetic foot care algorithm**				
SOP “Hygiene treatment of feet in diabetic patients”	600 (26.5%)	1663 (73.5%)	1382,837^a^	< 0.001
Study of foot vibration sensitivity in diabetic patients	308 (13.6%)	1955 (86.4%)	1507,472^a^	< 0.001
Study of foot vascularity in diabetic patients	252 (11.1%)	2011 (88.9%)	1367,247^a^	< 0.001
Study of foot tactile sensitivity in diabetic patients	250 (11.0%)	2013 (89.0%)	1373,473^a^	< 0.001
Feet sensitivity study in diabetic patients	281 (12.4%)	1982 (87.6%)	1278,569^a^	< 0.001
Foot examinations in diabetic patients	279 (12.3%)	1984 (87.7%)	1389,098^a^	< 0.001
**Emergency obstetric and gynecological care**				
OARIT Nurse management of severe pre-eclampsia and eclampsia	411 (18.2%)	1852 (81.8%)	1411,122^a^	< 0.001
Nurse management of a pregnant and postpartum woman on admission with hemorrhage	434 (19.2%)	1829 (80.8%)	859,932^a^	< 0.001

**Table 6:** Utilization of standard operating procedures in the management of patients with chronic heart failure, cerebral palsy and stroke.

**Standard operating procedures (S?P)**	***N* Yes**	**No**	**Chi-square**	***P*-value**
**Management of a patient with chronic heart failure**				
Measuring the patient's body weight in CHF	817 (36.1%)	1446 (63.9%)	174,830^a^	< 0.001
Self-care education for the cardiac patient	472 (20.9%)	1791 (79.1%)	768,785^?^	< 0.001
Determining daily diuresis and water balance in CHF	422 (18.6%)	1841 (81.4%)	889,775^a^	< 0.001
Counselling the CCN patient who requires palliative care	289 (12.8%)	1974 (87.2%)	1254,629^a^	< 0.001
Monitoring the ICF patient at the terminal stage	238 (10.5%)	2025 (89.5%)	1411,122^a^	< 0.001
Providing individual care for a terminally-ill cardiac patient	245 (10.8%)	2018 (89.2%)	1389,098^a^	< 0.001
Algorithm of actions by medical registrar when communicating with a patient	695 (30.7%)	1568 (69.3%)	336,778^a^	< 0.001
How to manage an aggressive or distressed patient	444 (19.6%)	1819 (80.4%)	835,451^a^	< 0.001
**Support for children with cerebral palsy**				
Assessment of hand use according to the MACS classification system in children with cerebral palsy	206 (9.1%)	2057 (90.9%)	1514,008^a^	< 0.001
CFCS Communication Function Classification System (CFCS) assessment in children with cerebral palsy	290 (12,8%)	1973 (87.2%)	1251,652^a^	< 0.001
Eating and Drinking Ability Classification System (EDACS) assessment in children with cerebral palsy	121 (5,3%)	2142 (94.7%)	1804,879^a^	< 0.001
**Rehabilitation after a stroke**				
Recovery of motor skills in patients with cerebral stroke	247 (10.9%)	2016 (89.1%)	1382,837^a^	< 0.001
Preventing injuries and falls	740 (32.7%)	1523 (67.3%)	270,919^a^	< 0.001
Cognitive rehabilitation of post-stroke patients	224 (9.9%)	2039 (90.1%)	1382,837^a^	< 0.001
Physical therapy for stroke patients	279 (12.3%)	1984 (87.7%)	1284,589^a^	< 0.001
Mindfulness therapy for stroke patients	208 (9.2%)	2055 (90.8%)	1507,472^a^	< 0.001
Physical activity evaluation of the patient	433 (19.1%)	1830 (80.9%)	862,399 ^a^	< 0.001

The letter “a” indicates statistically significant differences between the compared groups (*p* < 0.05).

The original version of this article has been updated.

